# Water use governance in a temperate region: Implications for agricultural climate change adaptation in the Northeastern United States

**DOI:** 10.1007/s13280-020-01417-6

**Published:** 2020-11-15

**Authors:** Rachel E. Schattman, Meredith T. Niles, Hannah M. Aitken

**Affiliations:** 1 School of Food and Agriculture, University of Maine, 5722 Deering Room 115, Orono, ME 04469-5722 USA; 2 Department of Nutrition and Food Sciences, University of Vermont, 350 March Life Sciences, Carrigan Wing, 109 Carrigan Drive, Burlington, VT 05405-0086 USA; 3 Charlotte, USA

**Keywords:** Agriculture, Climate change, Groundwater, Regulation, Surface water

## Abstract

Climate change and access to water are interrelated concerns for agriculture and other sectors, even in temperate regions. Governance approaches and regulatory frameworks determine who has access to water, for what purpose, and when. In the northeastern United States, water governance has historically been conducted by states through a combination of statutory guidance and common law. However, it is unclear what effect if current governance approaches will be sufficient for achieving resource conservation and equitable allocation in a changing climate. To provide insight into these issues, we conducted the first review of freshwater governance in the 12 states that comprise the U.S. Northeast. Specifically, we examine their heterogeneous approaches to surface and groundwater use, permitting and reporting, and scarcity provisions. Using agriculture as the sector of focus, we show through narrative review and quantitative analysis that change in the proportion of cropland that is irrigated in each state does not differ based on governance approach. We also suggest that future decades may bring regulatory shifts relevant to agriculture, changes in enforcement, increased competition between agriculture and other users, and greater potential competition between states for water resources. This case study raises the question: how should we prepare for the time when competition for, or degradation of, a resource surpasses the ability of existing governance mechanisms to ensure conservation and equitable distribution?

**Electronic supplementary material:**

The online version of this article (10.1007/s13280-020-01417-6) contains supplementary material, which is available to authorized users.

## Introduction

Freshwater resources are necessary for the long-term health and wellbeing of both human and ecological communities. However, supply and demand for are not always aligned, and the long-term ability to meet human water needs is a growing issue of concern (Tidwell et al. 2018). This is particularly true in agriculture, which is the largest user of water globally and accounts for two-thirds of water usage worldwide (Postel 2000). As the global population grows and demand for food increases, water use in agriculture has become a significant concern, even in historically water-rich and temperate regions. Agricultural water use is often a mix of surface and groundwater, depending on regional geography and climate. Global reliance on irrigation for crop production is increasing (Siebert et al. 2010). In many regions, tools for control of water resources have evolved from community-based systems to government management (Ostrom et al. 2003). Today, agricultural water use is frequently governed by complex policy networks, developed over long periods of time, and sometimes characterized by stakeholder disagreement and contention (Lubell et al. 2016).

The United States is one of the largest agricultural producers in the world. In the United States, governance frameworks for protecting and allocating freshwater for agricultural and other users are executed on a state-by-state basis. The rights of states to govern water use have been reinforced through federal legislation, including the Federal Power Act, the Clean Water Act of 1977, the Desert Lands Act of 1877, and the Reclamation Act of 1902 (Adler 2010). State-level governance strategies have developed over time, driven by both ecological changes and changes in freshwater demand, and include common law, regulation, and statutory guidance. In many U.S. states, water scarcity, contamination, competition between users, saltwater intrusion, and/or groundwater depletion have led to a shift away from governance through common law towards an integrated common law/regulatory approach. Regulatory mechanisms often used in water governance include water allocation, permitting, and required reporting. Both case law and regulations are rooted in statutory doctrines also adopted at the state level, for example *absolute dominion, public trust,* and *riparian rights* (Dellapenna 2013a).

Much of the focus on agricultural water use in the United States has been in the West, a region where limited supply and competing uses have led to decades-long conflicts. However, rates of irrigation are decreasing in western states while simultaneously increasing in regions east of the Mississippi (Stubbs 2016). In this research, we turn our attention from the West and explore the complex network of governance and policy for agricultural water use in the temperate region that is the Northeast. Specifically, we consider the implications of existing rules and regulations pertaining to agricultural water use, and ask how current governance frameworks will influence agriculture in the context of climate change. By examining trends in irrigation on agricultural land overtime in the Northeast, we explore the relationship between increasing acres of production grown under irrigation and contemporary regulatory frameworks.

Based on our review, we present two case studies (the states of Maine and Vermont) that illustrate the complexity of common law, regulation, and statutory governance applied to surface and groundwater resources. We summarize how these states address the defining characteristics of state-level water governance in the Northeast: withdrawal rules, reporting and permitting requirements, and scarcity provisions. Through these case studies, we show how foundational doctrines have guided governance through foundational legal decisions and contemporary regulatory frameworks. The case studies raise important questions about if and how heterogeneous water use regulations compound climate-related risks for agriculture. Our review raises issues that are likely to be salient in high-income temperate agricultural regions beyond the northeastern United States. This is particularly likely in regions where, like much of the Northeast, water use governance has not yet been tested by scarcity and conflict, but where climate change will lead to such challenges in coming decades.

## Agricultural water use in the Northeast

**Fig. 1:**
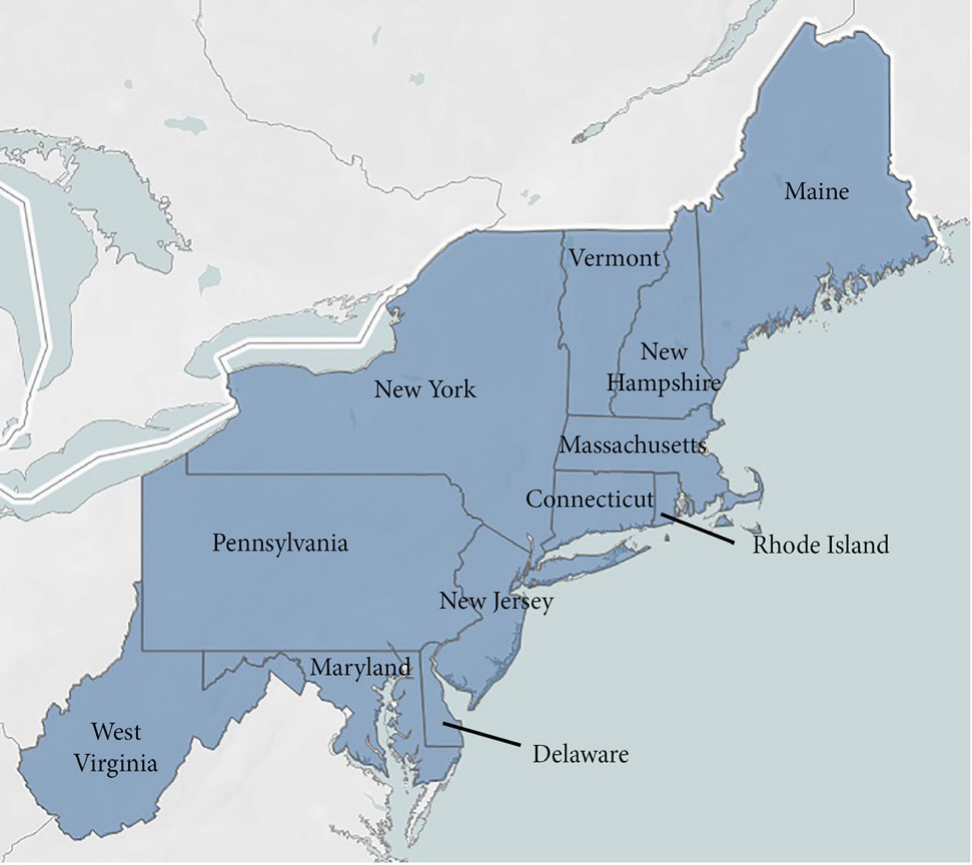
The Northeast region of the United States is composed of 12 states: Maine, New Hampshire, Vermont, New York, Pennsylvania, Massachusetts, Connecticut, Rhode Island, New Jersey, Maryland, Delaware, and West Virginia

**Fig. 2:**
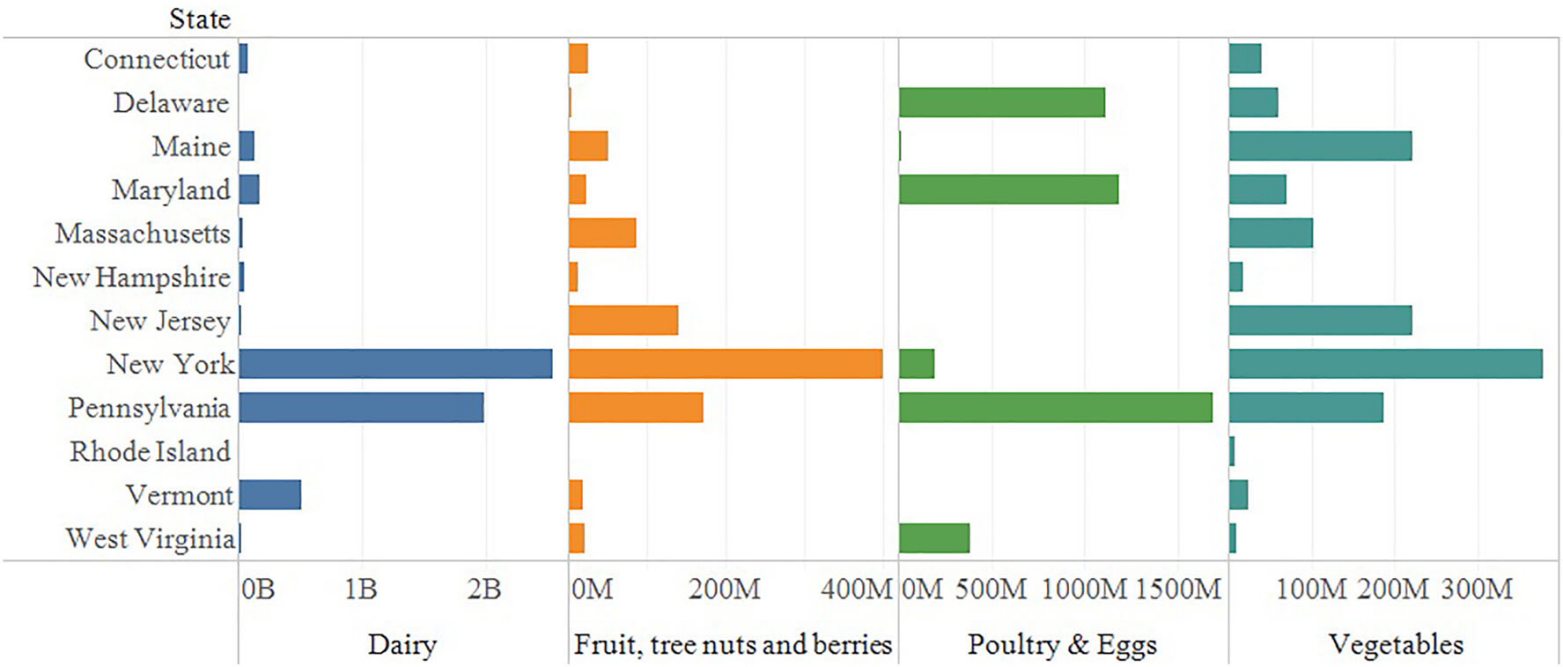
A selection of Northeast agricultural sales and numbers of farms reported in the 2017 USDA Agricultural Census (UDSA-NASS 2019). Figures on the *X* axis are USD annual gross sales; *B* billion; *M* million

The Northeast is a temperate, historically agrarian region with a diversity of agricultural sectors. Within the twelve states (Fig. 1) that comprise the region, farms produce vegetables, fruits, tree nuts, berries, nursery and greenhouse products, cattle and calves, dairy, poultry and eggs, and hogs (Fig. 2), accounting for 21.6 thousand million USD in annual sales (UDSA-NASS 2019). In 2017, the crop industry contributed over 8.8 thousand million USD annually and includes feedstock crops such as corn and hay, as well as specialty crops such as high value fruits and vegetables, berries, mushrooms, and ornamental nursery plants. The livestock industry was responsible for 12.8 thousand million USD, and includes cattle, poultry, and swine production, with dairy as the top agricultural commodity in the region. A few states are responsible for large percentages of Northeast production. Pennsylvania and New York combined represent 81% of dairy, 73% of cattle, 50% of nursery, 59% of fruit, and 42% of vegetable production in the region. Maryland and Pennsylvania combined represent 62% of the poultry and egg production, generating over 2.8 thousand million USD a year.

**Fig. 3:**
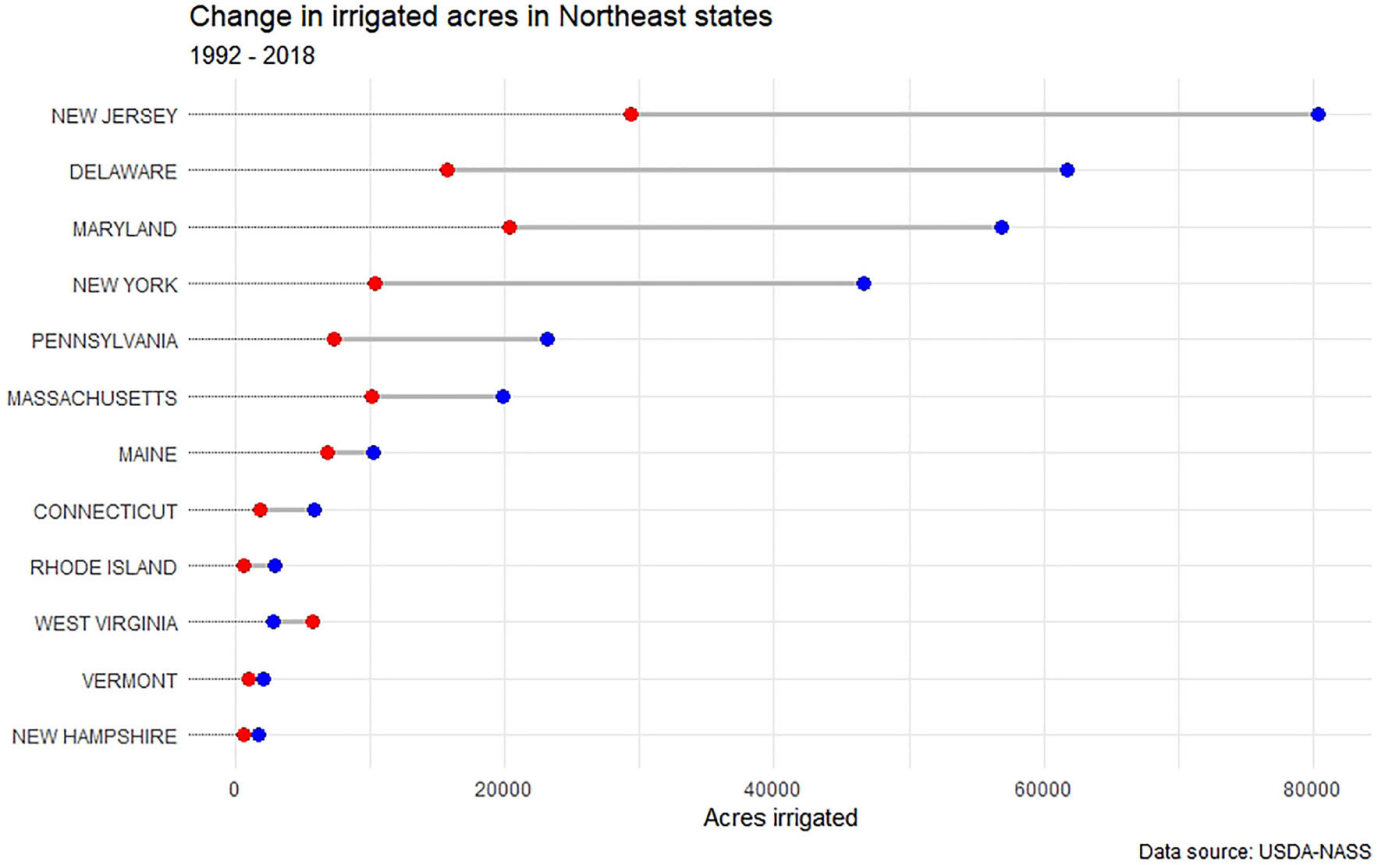
Change in irrigated agricultural acres in Northeast states. Red = 1992 acres; blue = 2018 acres

**Fig. 4:**
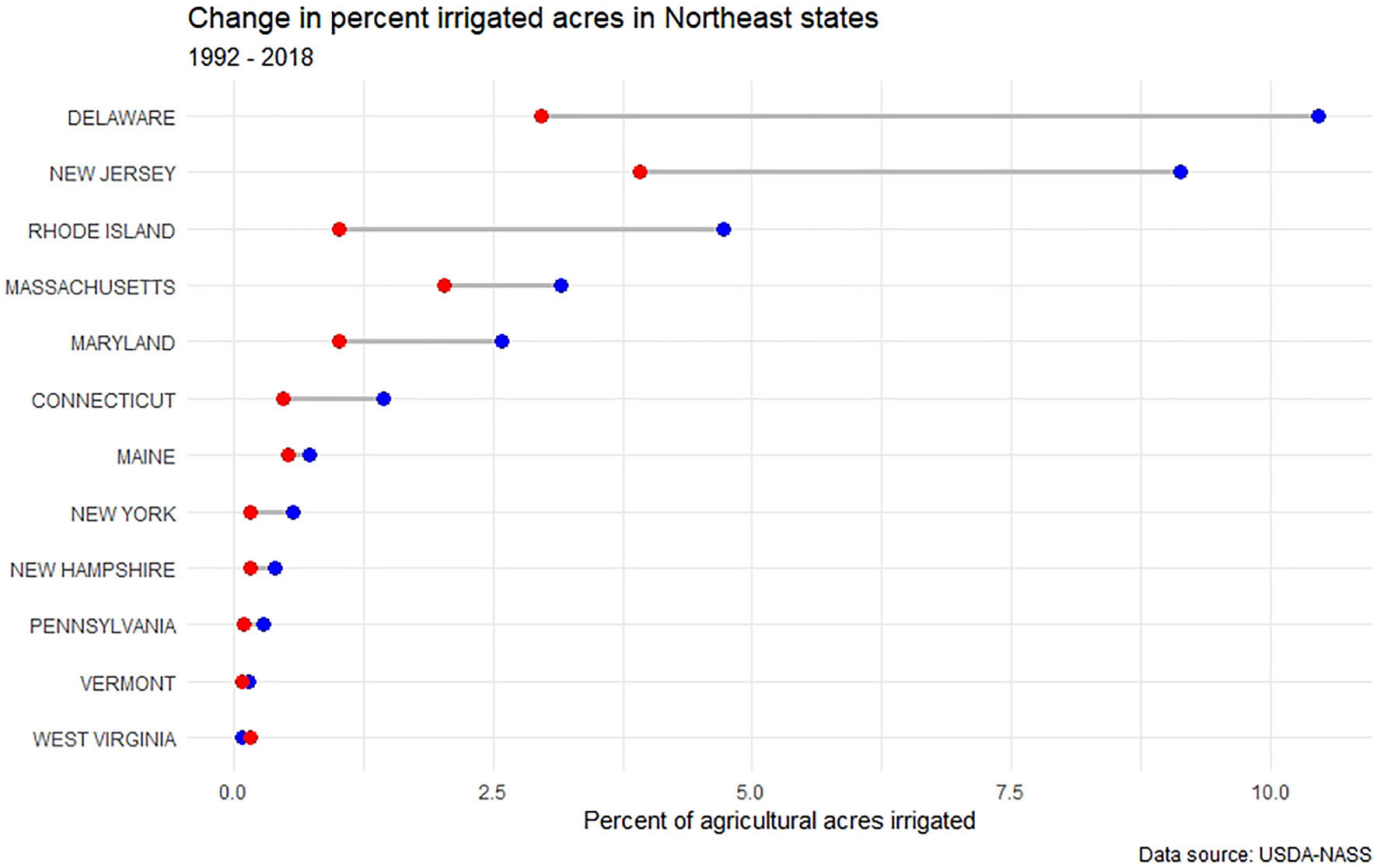
Change in the percentage of all agricultural land under irrigation in Northeast states. Red = 1992 acres; blue = 2018 acres

Historically, the Northeast has received sufficient annual rainfall to satisfy agricultural needs, though climate change may change this in some parts of the region in the future. In recent decades, the region has experienced increases in both high rainfall events and episodic drought, with current climate forecasts projecting continued increases for both dry and wet extremes (Horton et al. 2014). Agriculture in the Northeast relies on surface and groundwater for a variety of purposes, with irrigation representing a significant portion of the industry’s water use (Dieter et al. 2018; Hellerstein et al. 2019). Despite a projected increase in average yearly rainfall in coming decades (Dupigny-Giroux et al. 2018), access to irrigation water for crop production purposes remains critical to the success of agriculture in the region. In most Northeast states (with the exception of West Virginia), both the total number of irrigated agricultural acres and the proportion of total agricultural acres upon which irrigation is applied have grown (Figs. 3, 4). Delaware, New Jersey, and Rhode Island notably have the largest percentage increase in irrigated agricultural land in the region, while New Jersey, Delaware, Maryland, and New York have the largest total irrigated acres. It is likely that northeastern growers will continue to increase the amount of water they apply to crops in coming years due to shifts in precipitation patterns, with an increase in the frequency of dry late summers and early falls (Wolfe et al. 2018). Concurrently, growing populations along the Eastern seaboard and competition for water uses between agriculture, municipalities, and industry will increase the potential for conflict.

## Regional and state variation in water governance

Early attempts (prior to 1937) to govern water usage in the United States were inhibited by lack of knowledge on the part of lawmakers about the relationship between ground and surface water resources (Ausness 1983). Though scientific understanding of hydrogeology and withdrawal technology has dramatically progressed, governance approaches are not always well aligned with these developments. For example, most eastern states treat surface and groundwater sources as independent of one another, while in reality both are part of the same hydrogeological system (Dellapenna 2013b; Brown et al. 2019).

Approaches to water governance differ between U.S. states in the West and the East, driven by the relationship between water supply and demand, and competition between agricultural, industrial, municipal, and ecosystem users (Dellapenna 2013a). Western states, where water availability is low in proportion to need, often apply strict rules to limit water usage (Tidwell et al. 2018). It is clear that hydrological conditions, policy responses, and individual risk perceptions influence both agricultural producers’ support for water governance approaches and on-farm management decisions (Niles and Hammond Wagner 2019). Historically, eastern states have not contended with water scarcity to the same degree as western states as evidenced by less restrictive rules and regulations surrounding water extraction and wider variation of agricultural producers’ approach to water use efficiency practices (USDA-NASS 2019).

Governance of water resources begins with the foundational doctrine adopted by each state. In illustration of how foundational doctrines are used, consider the *riparian rights* doctrine as it is widely applied to surface water in eastern states. Under the guidance of this doctrine, users are allowed to access water so long as their use is considered reasonable. In cases where users’ rights and responsibilities are called into question, the doctrine requires courts to determine resource allocation based on a judicial interpretation of fairness with consideration for precedence (Swenson 1998). Adler (2010) notes that many *riparian rights* states do not often have provisions for determining which users should decrease usage during times of scarcity or drought, and conversely which users should be prioritized. In instances where users’ needs conflict with one another, he maintains that courts often hand down judgements preferential to large-scale users (Adler 2010).

**Fig. 5:**
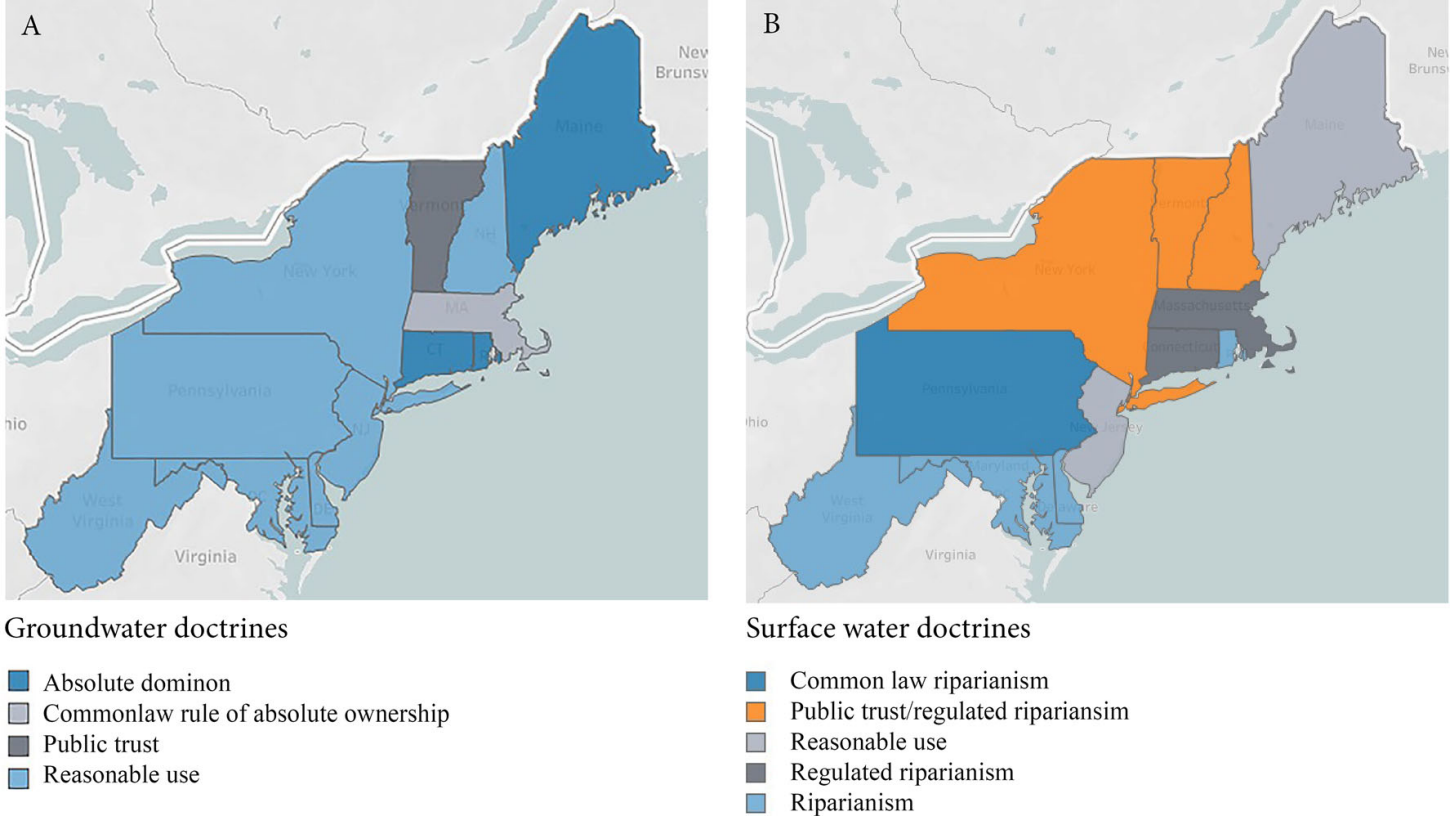
**a** Groundwater and **b** surface water doctrines used by Northeast states to guide withdrawal rules and regulations. For a full state-by-state summary, see Supplemental Materials, S1

In response to these conflicts, and the expensive and drawn out nature of judicial disputes, many states have applied doctrines to either partial or comprehensive regulatory frameworks. Components of such frameworks include permitting, reporting, and scarcity provisions. Statutory guidance may require states to inventory and monitor their water resources. With increased scientific understanding of surface and groundwater hydrology, many states have adopted statutory overlays and permitting systems that modify or supplement the original doctrines of their state, or use a hybrid of two doctrines. While states, even in regions with similar water resources, may operate under different guiding doctrines, it should be noted that rules and regulations that evolve from diverse doctrines may end up looking similar to each other. (A summary of doctrines and associated guiding principles is in Table 1, while Fig. 5 shows northeastern states that use each doctrine.)

**Table 1:** Overview of doctrines and guiding principles commonly applied to surface and groundwater governance in eastern states

Groundwater	
Absolute dominion	Also known as the rule *of capture*. Allows a landowner to use as much groundwater as they want without consideration of other landowners. Applies today in few Northeastern states, most notably in Maine where it was reaffirmed in 1999 (Tuholske 2008)
Reasonable use	Also known as the *American rule*. Requires allocated groundwater to be put to a reasonable use on the overlying tract of land it is taken from, and almost any amount or use of water can be considered reasonable depending on the state. Used in many states but some apply it in conjunction with other doctrines (Tuholske 2008)
Correlative rights	Permits landowners overlying an aquifer equal right to use the underlying water with preference given to those using the water on the overlying tract of land. All landowners over the shared aquifer are entitled to the common use of the water so long as they do not diminish another user’s ability to do so, even if the shared use of water diminishes the aquifer (Getches 1997; Tuholske 2008)
Public trust	Requires states to manage both the quality and quantity of groundwater for the benefit of its citizens. Establishes a framework that identifies groundwater as a vital resource that benefits all citizens and prioritizes public over private interest (Tuholske 2008)
Surface water	
Riparian rights	All riparian users have equal right to reasonable use of surface water, and upper proprietors of running water bodies cannot diminish the flow of water to a degree that would impact a lower proprietor. Water withdrawn but not consumed cannot be unreasonably detained or diverted and must be returned to the stream from where it was taken (Graham 1992; Dellapenna 2005; Kelley 2009)
Regulated riparianism	Statutory permitting implemented by a designated state agency (Dellapenna 2011)
Reasonable use	All riparian users may freely use the water so long as it does not unreasonably interfere with the use of other riparian owners (Graham 1992)

In illustration of how doctrines pertaining to surface and groundwater have been applied and modified through common law, and the resulting heterogeneity in existing rules and regulations, we highlight two eastern states: Vermont, which has adopted a *public trust* doctrine, and Maine, where *absolute dominion* for groundwater and *regulated riparian* rights for surface water had guided state-level regulations but where local negotiations between stakeholders have led to additional State programming, rules, and incentives for agricultural users. We also draw upon examples from Maryland and New Hampshire to show alternative approaches to regulations and programming, and demonstrate the variation of governance approaches across the Northeast region.

## Groundwater

The interactions between groundwater statutory guidance and common law decisions, and how these legal approaches apply to on-the-ground regulation, tend to be more complex and iterative than those pertaining to surface water. Historically, groundwater regulations in both Maine and Vermont were guided by *absolute dominion*, though how this doctrine was used to guide case law and agency enforced regulations differed. In Maine, *absolute dominion* has been both challenged and reaffirmed through legal action and statutory changes, in addition to the establishment of county and state-level commission and governing boards (for a historic timeline of these initiatives, see (Marvinney 2006). In 1987, the Maine legislature passed the Groundwater Protection Program, which established a cause of action against individuals who withdrew groundwater in excess of beneficial domestic use, or in instances when withdrawal interfered with preexisting beneficial domestic use (State of Maine 1987). This deviation from *absolute dominion* was challenged in court, and the doctrine reaffirmed in the *Maddocks v. Giles* decision (Maine Supreme Judicial Court 1999).

Additionally, local ecological and socio-economic contexts have driven county-level rules, programming, and incentive programs. Within Maine, three distinct contexts stand out: (1) in Downeast and Central Maine water users must balance agricultural needs with the protection of Atlantic Salmon (which are designated as a protected species); (2) agricultural users in Southern Maine must contend with limited supply and high development pressure, which intensifies competition between agricultural and municipal users; and (3) in highly agricultural Aroostook County, a limited opportunity for water resource development (e.g., pond construction) limits irrigation development (Harker 2012). The variable needs of each region have led to corresponding governance responses. For example, in Aroostook County where the majority of the state’s potato crop is grown, the Aroostook Water and Soil Management Board was established to conduct research on water use in anticipation of the Dickey-Lincoln Dam project (which was never completed), and to resolve conflicts between agricultural surface water users and other stakeholders. The board, partially funded by the U.S. Army Corp of Engineers, also conducted a multi-year effort to assess irrigation needs, institute a process to address water withdrawal complaints, and work with the Department of Environmental Protection (DEP) to address withdrawal limits, among other directives. Meanwhile, the *Downeast Rivers Water Use Management Plan* of 2000 was established to balance agricultural and conservation goals, specifically to protect Maine’s Atlantic Salmon population through farmer education and technical assistance, cost share assistance for development of farm ponds, and water resource monitoring (Marvinney 2006). These examples demonstrate the heterogeneity of governance and programming, even within a state governed by a single doctrine.

Vermont, like Maine, is an agricultural state where *absolute dominion* historically guided water use governance. *Absolute dominion* was preserved in Vermont State statute until 1985, when the State legislature passed the Vermont Groundwater Protection Act. This Act established the comparatively modern *correlative rights* doctrine (State of Vermont 2008) and called for the classification of the State’s groundwater. It should be noted that Vermont was the last state in New England to complete a groundwater resource assessment, a fact that has to some degree hindered the State’s ability to plan ahead for both increased demand and potential drought-related shortages (Mulholland 2006). From its earliest conception, the Act established a right of action if unreasonable harm resulted from “withdrawing, diverting or altering the character or quality” of groundwater, effectively abolishing the “common-law doctrine of absolute ownership,” while simultaneously building in limited protection for agriculture (State of Vermont 2008). While there has been limited litigation referencing this statute in Vermont, no court decisions have involved complaints concerning agricultural businesses or activities.

Despite the transition from *absolute dominion* to *correlative rights*, Vermont lawmakers were concerned about the doctrine’s influence (or lack thereof) on court decisions. For many years, the judicial system did not set precedent related to groundwater, leading some to maintain that Vermont remained under *absolute dominion* by default. Additionally, lawmakers were concerned that international treaties (such as the Transpacific Partnership and the North American Free Trade Agreement) or private international investors could establish use of the state’s groundwater under the *correlative rights* doctrine. These two factors ultimately led to the State’s decision in 2008, to replace *correlative rights* through expansion of the *public trust* doctrine (State of Vermont 2019). Vermont’s adoption of the *public trust* doctrine represented an important departure from all other guiding doctrines, which were and are primarily concerned with setting precedence among users. In contrast to other doctrines, *public trust* prioritizes the public good above the good of individual users. The *public trust* doctrine has been applied through Vermont common law, codifying its legitimacy in the regulatory environment (State of Vermont Superior Court 2011).

Compared to Maine, Vermont has seen little conflict and litigation concerning water use and agriculture, though irrigation withdrawals and other agricultural uses are regulated (though irregularly enforced). Limited conflict in Vermont around these issues is perhaps due to the relatively low demand agriculture places on groundwater resources: the most significant users of Vermont groundwater are private domestic and municipal users (Mulholland 2006), while agriculture is estimated to account for only two million gallons (4%) of Vermont’s estimated daily groundwater withdrawals (Walsh et al. 2011). Greater attention has been paid by far to the issue of Vermont’s water quality. This concern has been driven largely by a high load of legacy phosphorus associated with the State’s dairy sector, an industry that exports its products globally but must contend with animal waste on a regional scale (Wironen et al. 2018).

## Surface water

All northeastern states adhere in some form to *riparian rights* for surface water governance, either through *reasonable use* or *regulated riparianism*. However, the extent to which *reasonable use* is applied varies state to state (Dellapenna 2011). For example, surface waters in Vermont are held in the *public trust* and governed through a system of *regulated riparianism*. The Vermont Agency of Natural Resources, Department of Environmental Conservation (DEC) is charged to protect and maintain Vermont’s surface water quality, and administer the State’s water conservation policy (State of Vermont 2005; 2012a, b). Maine adheres to the *reasonable use* doctrine for surface water governance, and by doing so ensures that all riparian users may freely use the water so long as this use does not unreasonably interfere with the use of other downstream users (Kelley 2009).

Historically, there have been concerns that agricultural surface water use can lead to excessive drawdown of lakes, rivers, and streams, an issue addressed in Maine by setting limits on surface water withdrawals. In some cases, these limits have been developed by local and regional groups, such as those created by the Aroostook Water and Soil Management Board, in cooperation with State and Federal Agencies (Lombard et al. 2003). Because of the close relationship between *reasonable use* and *regulated riparianism* it is no surprise that Maine and Vermont have similar surface water management goals. However, the specifics of how these goals translate into rules and regulations illustrates how state-by-state (and sometimes county-by-county) determinations can lead to differences in the regulatory environment. These differences play out on the ground through reporting and permitting requirements, which are considered next.

## Permitting and reporting

**Fig. 6:**
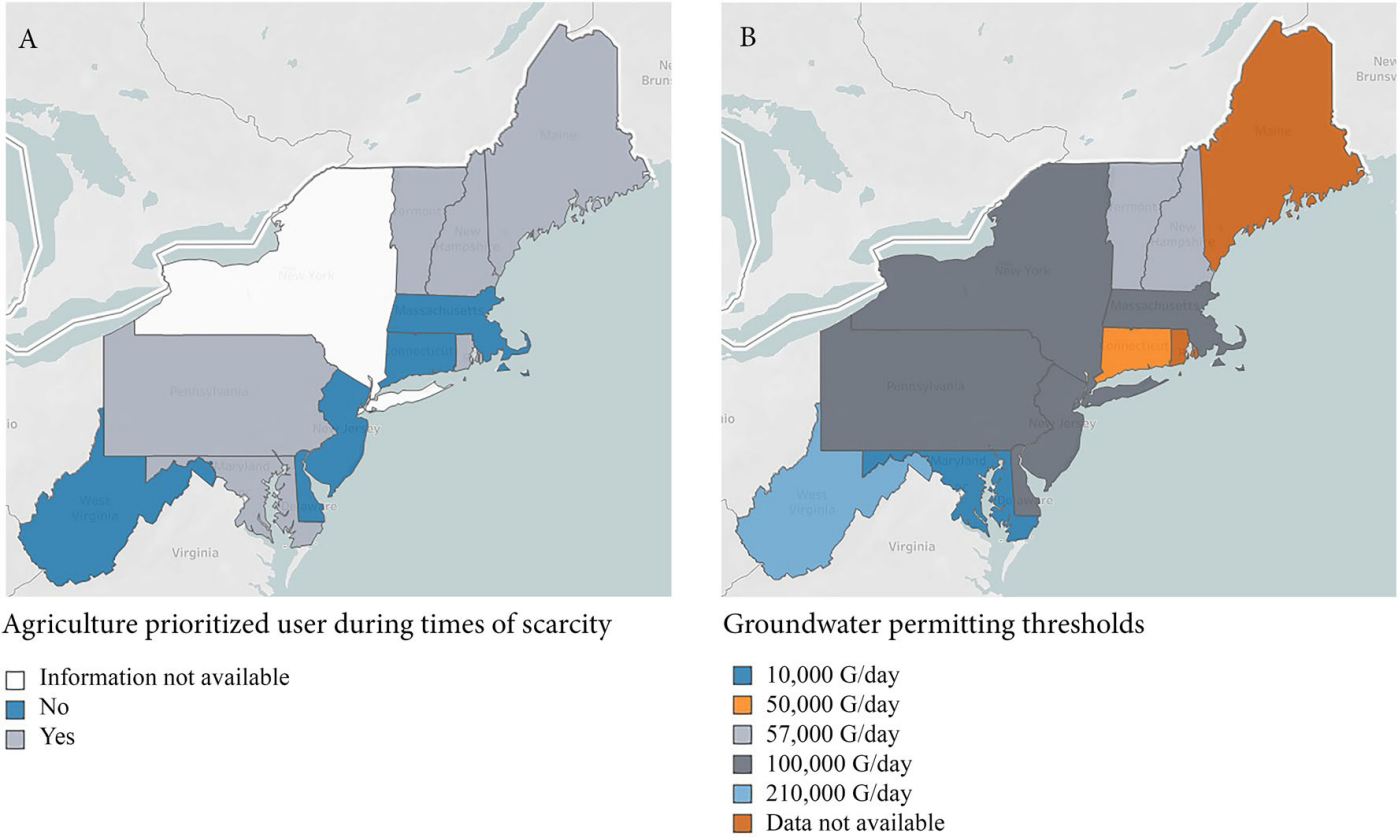
**a** States where agriculture is noted as a prioritized use during times of scarcity and drought; **b** groundwater permitting thresholds vary state-by-state. *G* gallons. For a summary of state-by-state requirements and reporting thresholds, see Supplemental Materials, S2

As with the application of doctrines, there is heterogeneity among Northeast states when it comes to withdrawal permitting and reporting guidelines. The degree to which agricultural users are exempt from existing guidelines or must comply with adjusted guidelines also varies. State-by-state approaches to these issues are summarized in Fig. 6, though it should be noted that some states apply permitting and reporting requirements at the county or watershed level. A summary for the 12 Northeast states is available in Table S2.

In Vermont, water withdrawals which alter or modify a riparian course, current or cross section of any stream may require a stream alteration permit (State of Vermont 2018). Negligible, or *de minimis,* withdrawals that do not alter the streamflow do not require a permit or reporting. Like Vermont, Maine has established minimum in-stream flows for protecting natural ecosystems and designated water uses (EPA 2007). In the past, threshold volumes for complying with the Maine rule began at 20 000 gallons (75 708 l)/day for a river, stream or brook, and 30 000 gallons (113 562 l)/week for lakes and ponds and change with the increase in the source size (State of Maine 2001). Since 2007, however, the State has created different requirements for agricultural water use based on the class of source water. Users must establish that in-stream flow or water level withdrawals are not excessive by complying with one of the following: “(1) a standard allowable alteration, (2) by a site-specific flow designation developed through an Alternative Water Flow or Alternative Water Level, or (3) as part of a new or existing regulatory permit” (State of Maine 2007). Vermont places the burden of establishing minimum in-stream flows at the time of pumping on the user. In Maine, the Department of Environmental Protection establishes the minimum in-stream flows, typically the August Mean Low Flow, by which the user must abide. The user must then work with the DEP to actually set the physical limit in the stream. However, many riparian agricultural stakeholders in Maine and Vermont are not knowledgeable about the rules, and they are not widely enforced across agricultural sectors. This raises the questions (1) whether periods of scarcity in the future would lead to increased enforcement by the Vermont DEC (potentially at the surprise of farmers), and Maine DEP and the Land Use Regulation Commission (LURC), and (2) how increased enforcement would affect agricultural operations.

In illustration of a different approach, the State of Maryland uses comprehensive permitting to control water use. The Maryland Department of the Environment (MDE) issues permits for all groundwater and surface water withdrawals greater than 10 000 gallons (37 854 l)/day. Applicants submit permit applications to the MDE and must provide satisfactory evidence that the proposed withdrawal will not jeopardize the State’s natural resources. For farms, this evidence typically includes a project map, a proposed water use, information about the irrigation system, crop or livestock type and acreage, and water sources. The State reserves the right to deny permits for uses deemed wasteful, dangerous, or detrimental to the public interest (State of Maryland 2014). By permitting water usage, states are enabled to not only monitor usage and natural conditions of ground and surface water, but also prioritize specific sectors (Ausness 1978). In Maryland’s hierarchy of uses, municipal public use ranks first with agriculture second, likely driven by the needs of a growing state population.

Both the *de minimis* approach (as adopted by Vermont), The Low Flow Rule and limited permit requirements in Maine, and the comprehensive permitting approach, as adopted by Maryland, show how states have moved away from strict common law governance governed by doctrines. Instead, regulatory approaches for water allocation have been widely applied across the eastern United States (Abrams 1990). The specific regulatory tools applied by the states are likely driven by the needs of a state or local region, evidenced by Maine’s approach to the different needs of three distinct regions of the state, Maryland’s thorough permit application process and hierarchy of use, and Vermont’s relative lack of conflict (and enforcement of current rules) around agricultural water withdrawal permits.

## Scarcity provisions

*Scarcity provisions* are preferences given to specific user groups during times of drought or reduced over ground flow, when water availability is limited for human and/or ecological needs. Under some regulatory frameworks, the management and designation of scarcity provisions are related to both permitting and reporting requirements. In the Northeast, Vermont, Maine, New Hampshire, and Maryland are examples of states that designate agriculture as either a first or second priority during drought (see Fig. 6).

In addition to establishing hierarchies of use, states have created programs designed to assist agricultural users to develop water resources. For example, scarce water resources during periods of drought have redoubled Maine’s attention to the needs of the agricultural sector. This is exemplified by policy and programmatic responses to the historic drought that occurred between 1999 and 2002. Between 2001 and 2002, agricultural losses associated with this drought totaled more than 32 million USD, with near total loss of the Maine wild blueberry crop (Lombard et al. 2003). In response, the State sled efforts to assist farms to develop ponds that would reduce their need to draw upon ecologically sensitive waters (e.g., low flow streams and wetlands). Simultaneously, ways to reduce farmers’ requirements to comply with federal and state wetland protection rules were explored. Additionally, funding was allocated to water use efficiency research in potatoes and blueberries, and in improving technical assistance (Marvinney 2006). These efforts demonstrate that, while scarcity provisions are important governance mechanisms, they are not the only tools available to states.

The relative efficacy of different governance mechanisms (e.g., scarcity provisions, incentive funding, technical assistance) to meet competing water use goals among state stakeholder groups remains unexplored in the Northeast. Likewise, the degree to which current rules, regulations, and statutes, including scarcity provisions, enable or obstruct necessary agricultural activities during times of drought remains largely untested. It is increasingly likely that such a test is forthcoming, considering the shifting precipitation regime in the region. It stands to reason that states that know what agricultural users need (through robust reporting programs) may be in a position to better protect those users during times of water scarcity and drought. However, this can be difficult to achieve as it is not uncommon for multiple agencies to have jurisdiction over different components of a state’s regulatory approach, and the cost of comprehensive permitting and oversight is likely high.

To complicate matters further, in many states, agencies with jurisdiction over water use report not having the capacity to fulfill their duties as assigned by state statute (Megdal et al. 2015). At a minimum, specificity and predictability are necessary when it comes to how water rights are allocated (Abrams 1990), the process for submitting permits and reporting water withdrawals, and any exemption or scarcity provisions. This enables users to plan on their level of water access during production periods, and tailor their activities accordingly (Ausness 1978). Better tracking (using meters instead of calculated pump outputs) is also necessary for assessing long-term water needs within states and regions (Levin and Zarrielo 2013).

## The relationship between agricultural water use and governance

As demonstrated through these case studies and our review of governance mechanisms, there is a great deal or heterogeneity between Northeast states. Considering the changes in agricultural water use in this region over the past several decades (see Figs. 3, 4), a reasonable question is whether or not this heterogeneity has a relationship to the increase in total and proportional irrigated acres? We used data collected thought the United States Department of Agriculture (USDA) Census of Agriculture and the Irrigation and Water Management Survey (formerly called the Farm and Ranch Irrigation Survey) to calculate the change in the percentage of irrigated farmland acres between 1992 and 2018 in Northeast states. A series of Chi-Square Goodness of Fit tests showed that the degree of change in proportional irrigated acres did not differ based on governance mechanism, including groundwater or surface water reporting rules, groundwater reporting or permitting thresholds, agricultural exemptions, agricultural prioritization during drought, or groundwater and surface water doctrines. Results are summarized in Table 2.

**Table 2:** Results of a series of Chi-Square Goodness of Fit tests that test the relationship between changes in the proportion of irrigated agricultural acres/all agricultural acres with various water governance mechanisms

Governance mechanism	*χ*^2^ (DF)	*p* value
Groundwater and/or surface water reporting rules are in place (yes/no)	12 (11)	0.364
Groundwater reporting threshold	72 (66)	0.286
Groundwater permitting threshold	60 (55)	0.299
Agriculture is exempt from permitting and reporting water use	12 (11)	0.364
Agriculture is prioritized during times of scarcity	24 (22)	0.347
Groundwater doctrine	36 (33)	0.330
Surface water doctrine	48 (44)	0.314

*DF* degrees of freedom

## Discussion and conclusion

Foundational doctrines have guided freshwater governance at the state level in the Northeast, giving shape to important legal decisions and contemporary regulatory frameworks. Statutory doctrines, common law, and regulatory approaches are interconnected and together compose a governance framework. States can alter their governing approach over time given public support, legislative will, and sufficient agency capacity. Through this review, we show that a variety of doctrines guide case law and set precedent, though contemporary regulatory frameworks vary based on local ecological and socio-economic contexts, and the ability of the state to integrate up-to-date hydrological science. Furthermore, we demonstrate the existing governance structures have no significant relationship to a shifting demand in agricultural irrigation water use. For example, through adopting the *public trust* doctrine, Vermont’s legislature has aligned its priorities around surface and groundwater management, in addition to requiring water resource and public good assessments. While most Northeast states have conducted assessment activities (e.g., groundwater mapping) prior to Vermont, and without adopting a doctrine resembling *public trust*, Vermont’s approach highlights how iterative governance can lead to outcomes that accommodate new knowledge, as well as new environmental and social contexts. Alignment of up-to-date scientific knowledge with freshwater governance approaches is critical to meeting the needs of a state or local region, as without it states are likely unable to anticipate how to best manage water resources (Mulholland 2006).

Considering this, how to best accomplish the integration of scientific information with governance is a salient topic. It has been shown that the application of scientific information, and in particular climate information, into long-term planning water resource decisions is best accomplished when that information is perceived by decision makers as being accurate, credible, reliable, timely, useful (Kirchhoff et al. 2013), and delivered by trusted organizations or individuals. There is opportunity to integrate climate data that could extend or add to scientific assessments currently used to assess water resources (i.e., well locations and percolation, location of bedrock) through development of models to predict sector by sector uses. By doing so, the scientific community can provide valuable information to state legislators and regulators, supporting them to meet state-by-state governance goals. While some efforts in this vein have been attempted in the Northeast, efforts have been limited by (a) the course nature of reported water use data (meaning the categories are not precise enough to make meaningful projections about future need), and (b) water use is typically measured using pump output calculations, which are notoriously inaccurate compared to metered assessments (Levin and Zarrielo 2013).

As demonstrated in the case studies, states have different approaches when it comes to resource assessment and ensuring that water withdrawals do not have detrimental effects on ecosystems or other users. Our review showcases three markedly different approaches to this issue: those that require users to calculate the rate below which extraction will have no negative effect, those where the State works with the user to develop a water management plan and work with State-mandated low flow limits, and those where permit proposals are evaluated and ultimately approved by a state agency. Assessment of surface water can be complex, and include characteristics such as stream classification system, stream flow, pumping frequency, watershed area, soil types, and the needs of local fish communities (Hamilton and Seelbach 2011). In cases where the user is required to establish the *de minimis* rate using only two variables (i.e., flow rate and watershed area), it is unlikely that the full effect of water withdrawal can be understood. Additionally, our experience with agricultural producers in the Northeast suggests that there is no widespread awareness of the requirement to establish the *de minimis* rate prior to pumping. Even in the case of widespread awareness, research on regulatory compliance shows that natural resource users are influenced by not only their awareness of the rules, but also calculated, normative, and social motivations, and their capacity to comply (Winter and May 2001). This helps to explain the apparent lack of relationship between water governance mechanisms and changes in the percentage of Northeast farmland that is irrigated.

Accountability, through inspections of practices or other means, is also an important motivator for farmer compliance with rules and regulations, assuming the cost of complying with regulations is less than potential fines associated with not complying (Herzfeld and Jongeneel 2012). This suggests that user compliance with freshwater protection requirements of various types should be further explored. The efficacy of state’s rules around withdrawal, reporting, and permitting may not be fully known until more severe and persistent periods of drought are experienced in the Northeast, and the ability of state agencies to monitor and enforce withdrawal limits is tested in the context of increased competition for freshwater resources. The findings from our review suggest that in historically water-rich regions, variations in accountability measures and other governance approaches do not necessarily lead to changes in agricultural water use, at least with the amount of heterogeneity that we currently observe in the northeastern United States. This does not mean, however, that current governance structures are suitable for addressing a rise in future resource demands.

In an era of climate change, states must balance competing water demands from various sectors within the context of increasing hydrologic uncertainty. Municipal demand has increased in the east due to population growth (Miller et al. 1997), and the likelihood for interstate conflict may increase if and when demand outpaces supply (Brown et al. 2019). While current governance mechanisms in the Northeast do not appear to have a direct relationship with the increased proportion of irrigation acres, it is conceivable that future demand will surpass these governance framework’s ability to equitably allocate and conserve water in the future. The challenges of managing freshwater resources at the state level are considerable, especially in contexts where the cost of monitoring and ecological resources is high, the demand for the resources is in flux, there is incongruence between the boundaries of the resources and regulated users (Dietz et al. 2003). Increasing competition for fresh water will likely require that states monitor their water resources carefully to ensure a balance between ecosystem and human wellbeing. Doing so will enable allocation that is in keeping with state goals and priorities.

Considering this, our review raises several important lines of inquiry. First, we have demonstrated that Northeast states apply a wide range of approaches to freshwater governance, but it is not known if these approaches enable agricultural users to make effective adaptation decisions in periods of water scarcity and drought. Similar questions are pertinent to other temperate and agricultural regions globally, as it is likely that many of them also rely on governance frameworks that have not yet been tested by the severity and frequency of drought forecasted by climate change models. Is it also possible that certain regulatory requirements obstruct agricultural producers’ ability to adapt to a changing climate, either through limiting water access or through confusing and unclear permitting requirements.

Second, the impact of prioritizing agriculture during times of drought and water scarcity is unclear, both on the agricultural community and on competitive user groups. Particularly in states that have adopted a *public trust* doctrine, the effect on each state’s interpretation of *public good* should be further examined in the context of water scarcity and preferential resource allocation. Scarcity provisions, technical assistance, incentive programs, and other mechanisms of state support should be evaluated based on their relative ability to protect different water use stakeholder groups (including agriculture) during periods of scarcity and drought, thereby enabling policy makers to better assess the risks of future climate change scenarios and allocate resources effectively.

Third, the potential for climate change to lead to more water scarcity in temperate regions, including the eastern United States, has been discussed in legal scholarship (for example, see Adler 2010). Despite this, little is known about how future water demands may compare to changes in water availability in agriculture. This is due, in part to the lack of detail required in reporting requirements in many Eastern states: in states that do require total usage reporting, crop by crop use is not required (Levin and Zarrielo 2013). There is also little documentation about how agricultural producers in eastern states think about water use regulations, their level of support for new policies in this domain moving forward, or how water governance approaches influence their management decisions, all of which are documented to be important factors related to policy support and management (Niles and Hammond Wagner 2019; Hammond Wagner and Niles 2020). This is an area of research that would serve lawmakers and regulators alike, as well as contribute valuable insights to the body of scholarship concerning the intersections between water policy and governance and climate change adaptation.

Finally, it is clear that in the Northeast, current governance mechanisms are heterogeneous with a range of reporting and permitting guidelines, scarcity provisions, approaches to prioritization, and underlying doctrines that guide them. This heterogeneity appears to have had little relationship to the increase in the proportion of farmland upon which irrigation is utilized in the Northeast. Considering the increase in acres irrigation along with climbing water demands in industrial and municipal sectors, two important questions arise: (1) at what threshold will demand overwhelm current governance approaches? and (2) how can we pre-emptily conserve and equitably distribute resources while avoiding conflict, prior to reaching this threshold? These questions can reasonably be posed, not only in regard to Northeast water governance, but in any region where a historically abundant critical natural resource is likely to become more competitive or degraded in the future.

There are two categories of governance mechanisms that states or regions in this situation could consider preemptively: (1) those that are designed to protect and equitably allocate scarce natural resources, and (2) those that are designed to decrease conflict across geopolitical boundaries. Returning to water as an example, we suggest that temperate regions look to regions with relative water scarcity, such as New Mexico’s Middle Rio Grande (MRG) watershed. In the MRG, water supply authorization of dam and reservoir operators (to execute both flood or water storage) and restrictive allocation strategies for water users are both utilized (Benson et al. 2014). Northeast states can learn from successes, but also reform efforts currently underway in regions like the MRG. Such reform efforts include exploration of more flexible adaptive management processes that are both responsive to changing regional conditions (Williams 2014) and grounded in specific statutory guidance (Biber 2014). Successful adaptive management of natural resources is characterized, in part, by governance frameworks that structurally allow for change, where governing bodies and managers have identified a suite of possible future trajectories, and where decision makers have the capacity to execute changes when appropriate (Benson et al. 2014).

There is also a need for state-level natural resource policy (including that pertaining to water) to be responsive and engage with cross-border governance issues. Discourse on water governance across political borders often includes scaling of governance (international, national, regional, local) (Woodhouse and Muller 2017), and the effect of scaled governance on both local decision making (Norman and Bakker 2009) and equitable access to natural resources. In response to concerns about future water scarcity and the conflicts that may stem from it, the United Nations (UN) has established water security as a human right, and through the UN World Water Assessment Programme has continually asserted that improved water governance is critical to securing this right (Woodhouse and Muller 2017). Despite widespread agreement on the aspirational vision of the Programme, the most recent version of the UN World Water Development Report has been critiqued for failing to acknowledge that unconstrained economic and population growth will inevitably surpass water supply (Boretti and Rosa 2019), and that stronger governance (as opposed to reliance on private and market drivers) are needed to ensure conservation and equitable access in an international context (Shah et al. 2018), and at the local level. To address persistent challenges associated with cross-border governance, we echo Armitage et al. (2015), who propose that improved water governance across geopolitical boundaries will require effective science–policy interactions facilitated through (a) recognition of the value of science in decision-making processes; (b) multi-stakeholder commitment and collaboration; (c) collaboration through group-learning processes, and by extension legitimacy of resulting decisions; (d) nurturing and engaging boundary organizations in which stakeholders (including scientists, policy makers, and citizens) can interact. These themes should be attended to in regions where limited resources necessitate negotiation and cooperation across political boundaries.

In summary, state-level freshwater governance in the Northeastern United States is notably heterogeneous, and it is unclear to what degree this heterogeneity will enable or inhibit agriculture to adapt to a changing climate. It is well established that shifting precipitation patterns will have an impact on agriculture in temperate regions globally, including the Northeast region of the United States, and that this change will present significant challenges to agricultural producers. Of great importance is whether agricultural producers will have access to water resources during periods of drought and scarcity. Water use governance is based in historical doctrine and precedence, and yet must be responsive to changing socio-economic contexts. Therefore, we believe there is an opportunity for states in temperate regions to proactively assess their governance approaches, including their withdrawal regulations, permitting, and reporting requirements, and scarcity provisions. Doing so could help ensure that the effects of a changing climate do not undermine our ability to produce food, fiber, and fuel in agriculturally important regions worldwide. More broadly, water in the U.S. Northeast is an example of a natural resource that has been historically plentiful, and has been governed as such. Our review raises important questions about what happens when competition for such resources surpasses governance mechanisms’ ability to conserve them or allocate them equitably. This question could be asked in the context of any natural resource that transitions from non-rival to rival, and thus necessitates an adaptive and responsive set of governance mechanisms.

## Electronic supplementary material

Below is the link to the electronic supplementary material.

Supplementary material 1 (PDF 126 kb)
