# Supplementary material for: Water use governance in a temperate region: Implications for agricultural climate change adaptation in the Northeastern United States
Source: Ambio. 2020 Nov 15;50(4):942–55. doi: 10.1007/s13280-020-01417-6 (PMC7982366; doi:10.1007/s13280-020-01417-6)
Supplement: Supplementary file 1 — Supplementary material 1 (PDF 126 kb) [file 13280_2020_1417_MOESM1_ESM.pdf]

Electronic supplementary material

This supplementary material has not been peer reviewed

Title: Water use governance in a temperate region: Implications for agricultural climate change adaptation in the Northeastern United States

Authors: Rachel E. Schattman, Meredith T. Niles, & Hannah M. Aitken

Table S1: *Summary of water governance doctrine and regulatory approaches in 12 Northeast states*

	**Groundwater**	**Surface water**
**Connecticut**	*Absolute Dominion:* Agriculture is not explicitly prioritized during times of scarcity.	*Regulated riparianism:* Agriculture is not explicitly prioritized during times of scarcity.
**Delaware**	*Reasonable Use:* Rule applies to groundwater, held in the public trust.	*Riparianism:* Agriculture is not explicitly prioritized in times of scarcity.
**Maine**	*Absolute dominion:* Agriculture is a prioritized use during times of scarcity.	*Reasonable use:* Agricultural producers must not withdraw surface water past the point where it interferes with established flow and water level requirements.
**Maryland**	*Reasonable use:* The landowner owns the groundwater below the surface. During times of scarcity, agricultural users are prioritized.	*Riparianism:* Agriculture is prioritized in times of scarcity, but must document flow and not overdraw surface water.
**Massachusetts**	*Common law rule of absolute ownership:* Agriculture is not explicitly prioritized during times of scarcity.	*Regulated riparianism:* Agriculture is not explicitly prioritized during times of scarcity.
**New Hampshire**	*Reasonable use:* Agriculture is not a prioritized use.	*Regulated riparianism and public trust:* Registered water users (including farms) must take measures to protect surface water supplies during periods of scarcity. Water use may be limited during these periods, but agricultural uses are prioritized.
**New Jersey**	*Reasonable use:* Restrictions apply to users during times of drought, or in areas where the water resource has been significantly depleted or compromised. Agricultural users are not exempt from restrictions.	
**New York**	*Reasonable Use:* The state is the owner of groundwater resources. Courts have recognized that over-pumping groundwater can interfere with surface water flow and access.	*Riparianism and public trust:* The landowner whose property bounds a body of water have rights to that water, so long as the use is reasonable, and must not interfere with the use by other riparian owners of that same water.
**Pennsylvania**	*Reasonable Use:* Rule applies to percolating groundwater. PA law does not explicitly address underground subterranean streams. In times of scarcity agriculture is prioritized third after domestic uses and navigable uses.	*Common Law Riparianism:* The right is usufructuary and does not allow for diversions of water to be used outside of the adjacent riparian tract of land.
**Rhode Island**	*Absolute dominion:* Priority given to commercial agricultural producers who have an approved water withdrawal management plan.	*Riparianism:* Priority given to commercial agricultural producers who have a water withdrawal management plan approved by the DEM.
**Vermont**	*Public Trust:* Agriculture is a prioritized use during times of scarcity.	*Regulated riparianism and public trust:* Agriculture is not prioritized use during times of scarcity.
**West Virginia**	*Reasonable Use:* Rule applies to groundwater.	*Riparianism:* Agriculture is not explicitly prioritized in times of scarcity.

Table S2: *Summary of reporting, permit, and registration requirements for 12 Northeast states*

	Reporting requirements	Permit or registration requirements
**Connecticut**	*Groundwater* and *surface water* must be reported annually if withdrawals are > 50000 gallons (189271 liters)/day.	*Groundwater* and *surface water* must be permitted if withdrawals are > 50000 gallons (189271 liters)/day.
**Delaware**	*Groundwater* and *surface water* reporting required. Agricultural water users only need to submit irrigation water use records for withdrawals between March and November.	*Groundwater* and *surface water* permits are required if withdrawals are > 50000 gallons (189271 liters)/day. An additional permit is required if withdrawals are > 100000 gallons (378541 liters)/day within the jurisdiction of the Delaware River Basin.<br><br>Agricultural irrigation wells may automatically receive permits in certain circumstances.
**Maine**	*Groundwater* reporting is required for withdrawals >50,000 gallons/day. Agricultural uses are exempt.<br><br>*Surface water* withdrawals presently have no reporting requirements.	*Groundwater* withdrawals require permits. Agricultural uses are exempt.<br><br>*Surface water* withdrawals for any use (including agricultural purposes) require permits.<br><br>Agriculture is designated as a high-priority user.
**Maryland**	*Groundwater* and *surface water* withdrawals must be reported if agricultural users qualify for a permit.	*Groundwater* and *surface water* permits required for withdrawals > 10000 gallons (37854 liters)/day.
**Massachusetts**	*Groundwater* and *surface water* withdrawals must be reported if agricultural users qualify for a permit.	*Groundwater* and *surface water* permits required for withdrawals > 100000 gallons (378541 liters)/day on average over a 3-month period.<br><br>Exceptions apply to some cranberry growers.
**New Hampshire**	*Groundwater* and *surface water* agricultural water withdrawals > 20000 gallons (75708 liters)/day averaged over 7-days, or 600000 gallons (2271247 liters) in a 30-day period must be reported. Seasonal water users must report monthly usage. If farms use water year-round, reports must be submitted quarterly.	*Groundwater* withdrawals > 57600 gallons (218039 liters)/day must be reported.<br><br>*Surface water* withdrawals must be reported unless they are intermittent and are not > 20000 gallons (75708 liters)/day averaged over a 7 day-period or over 600000 gallons (2271247 liters) in a 30-day period.
**New Jersey**	*Groundwater* and *surface water* monthly usage reports are required of users with approved "water usage certifications."<br><br>For agricultural users, reports include the irrigated crops and the number of acres planted.	*Groundwater* and *surface water* withdrawal permits are required if a user has the capacity to withdraw > 100000 gallons (378541 liters)/day.

**New York**	*Groundwater* and *surface water* withdrawals for agricultural use must be reported if withdrawals are > 100000 gallons (378541 liters)/day in any 30-day period.	*Groundwater* and *surface water* permits required for withdrawals > 100000 gallons (378541 liters)/day.<br><br>Agricultural users are required to register if withdrawals are > 100000 gallons (378541 liters)/day in any 30-day period.
**Pennsylvania**	*Groundwater* and *surface water* withdrawals for agricultural use must be reported if withdrawals are > 100000 gallons (378541 liters)/day in any consecutive 30-day period.	*Groundwater* and *surface water* permits required for withdrawals > 100000 gallons (378541 liters)/day. In designated groundwater protection areas, permits are required for withdrawals > 10000 gallons (37854 liters)/day.
**Rhode Island**	*Groundwater* and *surface water* agricultural water withdrawals do not need to be reported.	*Groundwater* and *surface water* agricultural water withdrawals do not need to be permitted.
**Vermont**	*Groundwater* withdrawals must be reported > 20000 gallons (75708 liters)/day averaged over a calendar month.<br><br>Agricultural uses are exempt with exception of large scale withdrawals exceeding 57600 gallons (218039 liters)/day averaged over one month.	*Groundwater* withdrawal permits are required for users withdrawing > 57600 gallons (218039 liters)/day. Agriculture is exempt from the permitting process.<br><br>*Surface water* withdrawals for any use (including agricultural purposes) above the *de minimis* rate require permits. Agriculture is not exempt.
**West Virginia**	*Groundwater* and *surface water* reporting is required if withdrawals > 300000 gallons (1135624 liters) in any 30-day period.	*Groundwater* and *surface water* permits are required for natural gas operators developing horizontal wells that use > 210000 gallons (37854 liters).
